# *In Vitro* Metabolism of 20(R)-25-Methoxyl-Dammarane-3, 12, 20-Triol from *Panax notoginseng* in Human, Monkey, Dog, Rat, and Mouse Liver Microsomes

**DOI:** 10.1371/journal.pone.0094962

**Published:** 2014-04-15

**Authors:** Xiangrong Zhang, Ji Zhang, Wei Li, Li Liu, Baoshan Sun, Zhenghong Guo, Caihong Shi, Yuqing Zhao

**Affiliations:** 1 School of Traditional Chinese Materia Medica, Shenyang Pharmaceutical University, Shenyang, Liaoning, China; 2 Key Laboratory of Drug Metabolism and Pharmacokinetics, China Pharmaceutical University, Nanjing, Jiangsu, China; 3 Key Laboratory of Research and Design of “Drug Targets Based on the Ministry of Education”, Shenyang Pharmaceutical University, Shenyang, Liaoning, China; Texas Tech Univ School of Pharmacy, United States of America

## Abstract

The present study characterized *in vitro* metabolites of 20(R)-25-methoxyl-dammarane-3*β*, 12*β*, 20-triol (20(R)-25-OCH_3_-PPD) in mouse, rat, dog, monkey and human liver microsomes. 20(R)-25-OCH_3_-PPD was incubated with liver microsomes in the presence of NADPH. The reaction mixtures and the metabolites were identified on the basis of their mass profiles using LC-Q/TOF and were quantified using triple quadrupole instrument by multiple reaction monitoring. A total of 7 metabolites (M1–M7) of the phase I metabolites were detected in all species. 25(R)-OCH_3_-PPD was metabolized by hydroxylation, dehydrogenation, and O-demethylation. Enzyme kinetic of 20(R)-25-OCH_3_-PPD metabolism was evaluated in rat and human hepatic microsomes. Incubations studies with selective chemical inhibitors demonstrated that the metabolism of 20(R)-25-OCH_3_-PPD was primarily mediated by CYP3A4. We conclude that 20(R)-25-OCH_3_-PPD was metabolized extensively in mammalian species of mouse, rat, dog, monkey, and human. CYP3A4-catalyzed oxygenation metabolism played an important role in the disposition of 25(R)-OCH_3_-PPD, especially at the C-20 hydroxyl group.

## Introduction

**Figure 1:**
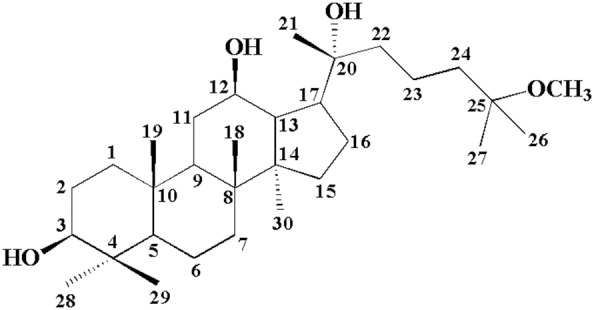
Chemical structure of 25 (R)-OCH_3_-PPD.

Panax ginseng, a traditional herbal medicine used in the Eastern Asia for more than 2000 years, is presently being used worldwide as one of the most common complementary alternative medicines [1]. Ginseng, the root of Panax ginseng, has various health benefits, ranging from overcoming fatigue to treating severe cardiac and cancer problems [2]–[5]. 25-methoxydammarane-3, 12, 20-triol (25-OCH_3_-PPD) was a novel dammarane-type triterpene sapogenin that was first reported by Zhao [6], [7], from the leaves of *P.notoginseng.* 25-OCH_3_-PPD exerts the strongest activity among any of the known ginsenosides tested for cytotoxic effects [8]. It is an effective inhibitor of cell growth and proliferation and inducer of apoptosis and cell cycle arrest. 25-OCH_3_-PPD had significant, dose-dependent effects on apoptosis, proliferation, and cell cycle progression. Preclinical data indicate that 25-OCH_3_-PPD is a potential therapeutic agent against prostate cancer [9] and human breast cancer through down-regulating MDM2 [10]. 25(R)-OCH_3_-PPD (Figure 1) showed anti-lung cancer activity by activation of p38 MAPK pathway and generation of reactive oxygen species [11].

20(S) and 20(R) forms of 25-OCH_3_-PPD are stereoisomers of each other that depend on the orientation of the C-20 hydroxyl. During the preclinical evaluation, we found that 25(S)-OCH_3_-PPD could be absorbed with an absolute bioavailability of 19.7% ±7.6% (10 mg/kg) in rats [12]. 25-OCH_3_-PPD was converted to 25-OH-PPD in rats after oral gavage or iv injection. 25(S)-OCH_3_-PPD was metabolized to form active 25-OH-PPD after oral and intravenous administration. Plasma level of 25-OH-PPD was greater than that of 25-OCH_3_-PPD. However, 25-OH-PPD was less active than parent compounds *in vitro* [6]. The pharmacokinetic profiles of 25(R)-OCH_3_-PPD and 25(S)-OCH_3_-PPD were different. 25(R)-OCH_3_-PPD was absorbed less than that of 25(S)-OCH_3_-PPD in rats [13].

It is important to examine whether or not 25(R)-OCH_3_-PPD was metabolized by drug-metabolizing enzyme(s) in liver microsomes and then attempted to elucidate human P450 isoform(s) responsible for the biotransformation of 25(R)-OCH_3_-PPD. The study of hepatic microsomal-mediated metabolism will be of great significance for understanding of their systematic disposition and of 25(R)-OCH_3_-PPD-drug interaction and the mechanisms involved.

The objectives of the present study were as follows: 1) to characterize the structures of oxidative metabolites of 25(R)-OCH_3_-PPD, 2) to examine the difference of metabolism of 25(R)-OCH_3_-PPD by rodent (mice and rats) and nonrodent (dogs and monkeys) animal species hepatic microsomes and 3) to identify the cytochrome P450 (P450) and non-P450 enzymes responsible for 25(R)-OCH_3_-PPD biotransformation.

## Materials and Methods

### Reagents

25(R)-OCH_3_-PPD and 25(R)-OH-PPD (99.0% pure) were supplied by Shenyang Pharmaceutical University (Liaoning, P. R. China). Pooled human liver microsomes (HLM), male cynomolgus monkey liver microsomes (CyLM), male beagles [dog liver microsomes (DLM)], male Sprague-Dawley rats [rat liver microsomes (RLM)], and male CD-1 mice [mouse liver microsomes (MLM)], as well as recombinant P450 enzymes CYP3A4 were purchased from Research Institute for Liver Diseases (Shanghai) Co., Ltd. (Shanghai, China). NADPH, NAD^+^, glucose-6-phosphate (G-6-P) and glucose-6-phosphate dehydrogenase (G-6-PDH) were purchased from Sigma-Aldrich (St.Louis, MO). All solvents used for high-performance liquid chromatography (HPLC) were of HPLC grade (Merck, Darmstadt, Germany). Chemical inhibitors, ketoconazole (Ket), α-naphthoflavone (Naph), sulfaphenazole (Sul), quercetin (Que), quinidine (Qui),diethyl dithiocarbamate (DDC) and ticlopidine (TP) (purity >99%) were purchased from Sigma-Aldrich (Shanghai, China). Purified water was generated with a Milli-Q Gradient system (Millipore Corporation, Molsheim, France).

### Chromatography of 25(R)-OCH_3_-PPD and Metabolites

The Agilent 1200 HPLC system was equipped with a reversed-phase column (Atlantis T3C_18_, 2.1×150 mm i.d., 5 µm; Waters CA) protected by a 4.0×3.0 mm i.d. Security Guard (5 µm) C_18_ guard column (Phenomenex, Torrance, CA). Mobile phases A and B consisted of 0.1% (volume fraction) formic acid in water and methanol, respectively. Gradient elution at a flow rate of 0.2 ml/min was with the following program: initial 70% B for 3 min, increased to 95% in 14 min, maintaining this condition for 8 min, ramping back down to 70% B in 1 min and holding at this condition for a further 4 min, to give a total runtime of 30 min.

### Mass Spectrometry

The initial screening of the compounds’ metabolites and accurate mass measurements was carried out using Agilent liquid chromatography quadrupole – time –of–flight mass spectrometer (LC-Q/TOF) 6520 mass spectrometer. A generic positive electrospray ionization method was used for all substrates and metabolites. The capillary voltage was 3500 V, cone voltage 20 V, and desolvation and source temperatures 260 and 150°C, respectively. Nitrogen was used as the desolvation and cone gas with flow rates of 900 and 300 L/h. All data acquired were processed using Mass Hunter Qualitative Analysis Software (B.04.00, Agilent).

The quantification (multiple reaction monitoring, MRM) and fragmentation measurements were performed with Shimadzu LC-MS/MS-8030 triple quadrupole instrument following positive mode electrospray ionization (ESI). The capillary voltage was 4500 V, and desolvation and source temperatures 250 and 150°C, respectively. The collision gas was argon with a CID gas cell pressure of 2.0×10^3 ^mbar. Nitrogen was used as the drying and nebulizing gas with flow rates of 90 and 15 L/h. The data generated was processed using Shimadzu LabSolutions version 5.

### Metabolism by Human, Monkey, Dog, Rat, and Mouse Liver Microsomes

25(*R*)-OCH_3_-PPD were incubated in hepatic microsomes to examine the potential P450-mediated metabolism. Stock solutions of 2 mM 25(R)-OCH_3_-PPD was prepared in methanol. The final methanol concentration in the incubation was 2.5% (v/v). The liver microsomes were carefully thawed on ice before the experiment.

For NADPH dependent oxidative metabolism study, the reaction media contained 1 mg/ml of microsomal proteins, 10 mM G-6-P, 10 mM MgCl_2_, 1 U/ml G-6-PDH, 50 µM concentration of 25(R)-OCH_3_-PPD in potassium phosphate buffer (pH 7.4; 0.1 M) in a total volume of 200 µl. Negative control incubations containing no NADPH-regenerating system were conducted. All incubations were performed at 37°C in a shaking water bath for 60 min.

After 5 min preincubation at 37°C, the incubation reactions were initiated with the addition of microsome proteins. After undergoing incubation for 60 min, the reactions were terminated with an equal volume of ice-cold acetonitrile and 10 µl diazepam (5 µg/ml, internal standard) was added. This sample was vortex-mixed and centrifuged at 14,000 *g* for 5 min. The supernatant was transferred into a glass tube, evaporated to dryness under a stream of nitrogen at 40°C. The residue was reconstituted in 100 µl of methanol with 0.1% formic acid, centrifuged 10 min at 18000 rpm and then an aliquot 5 µL of the supernatant was directly injected onto the HPLC-MS system for quanlitative or quantitative analysis.

### Chemical Inhibition Study in Human Hepatic Microsomes

The chemical inhibition study was performed by adding each of the specific inhibitors of P450 enzymes into the incubation of 25(R)-OCH_3_-PPD (3 or 10 µM) in 0.25 mg/ml HLM incubation systems containing an NADPH-regenerations system as described above. The chemical inhibitors and concentration were selected on the basis of previous reports and are as follows: α-naphthoflavone (10.0 µM) for CYP1A2, quercetin (10 µM) for CYP2C8, sulfaphenazole (10.0 µM) for CYP2C9, ticlopidine (5 µM) for CYP2C19, quinidine (10.0 µM) CYP2D6, diethyl dithiocarbamate (20 µM) for CYP2E1, and ketoconazole (0.1, 0.25, 0.5, 1 and 5 µM) for CYP3A.

The inhibitors were dissolved in dimethyl sulfoxide, and the final concentration of dimethyl sulfoxide in the incubation was 1% (v/v). The final incubation volume was 200 µl. Microsomes were preincubated for 5 min with a NADPH-regenerating system at 37°C, the reactions were initiated with the addition of 25(*R*)-OCH_3_-PPD and inhibitor. The reactions were terminated with an equal volume of ice-cold acetonitrile after 30 min of incubation. Parallel incubations without chemical inhibitors served as control. Samples were prepared as mentioned above to measure the concentration of parent and the metabolites by HPLC-MS/MS analysis.

### Recombinant Enzyme Incubations

25(R)-OCH_3_-PPD (3, 10 µM) was incubated in duplicate at 37°C for 30 min with a panel of recombinant human P450 enzymes CYP3A4, at 4 pmol/ml. NADPH-regenerations system was the same as part of metabolism by human, monkey, dog, rat, and mouse liver microsomes. Samples were prepared as previously described and an aliquot 5 µl was subjected to HPLC-MS/MS analysis to measure the metabolites formed. Parallel incubations without NADPH-regenerations system served as control.

### Enzymatic Kinetics of 25(R)-OCH_3_-PPD Metabolism by HLM

25(R)-OCH_3_-PPD was incubated with 0.25 mg/ml HLM at eleven different concentrations (1, 2, 3, 4, 5, 7.5, 10, 15, 20, 30, 50 µM). Reactions were kept at 37°C for 30 min, and then terminated with 200 µl of ice-cold acetonitrile containing IS. HPLC-MS/MS analyses of samples were the same as mentioned above. The apparent *K*_m_, *V*_max_ values were estimated by nonlinear regression from the Lineweaver-Burk plots based on typical Michaelis-Menten equation. The intrinsic clearance was calculated as *V*_max_/*K*_m_. Data are expressed as the mean ± S.D. of triplicate experiments.

### Statistical Analysis

All of the data reported represent the mean ± standard deviation. All experiments were conducted at least twice. The t-test was used for the statistical data analysis and the level of significant was set at *p*-value of 0.05.

## Results

### Mass Spectral Properties of 25(R)-OCH_3_-PPD and Metabolites

**Figure 2:**
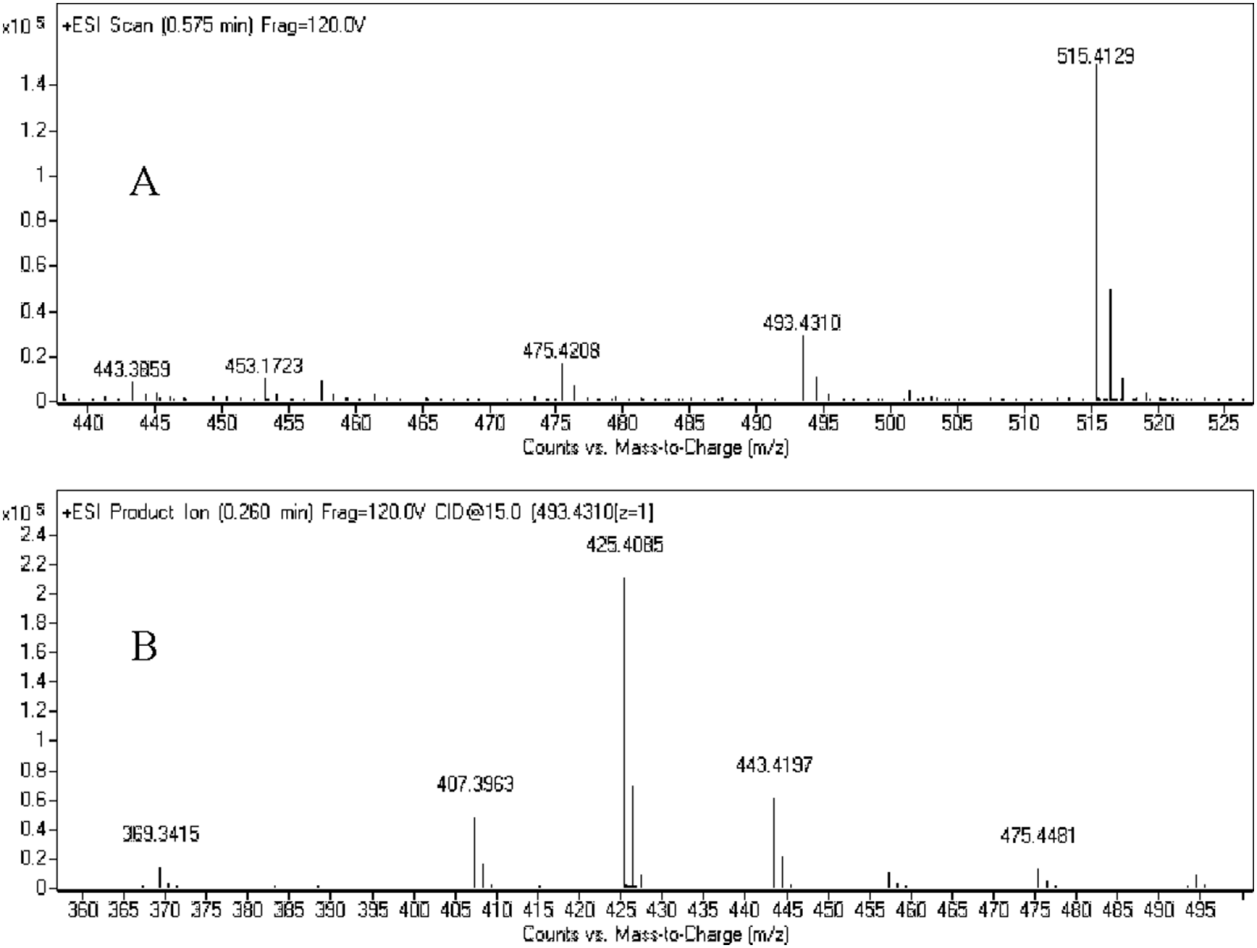
Full-scan mass spectrum (A) and MS^2^ spectrum (B) of 25 (R)-OCH_3_-PPD.

**Figure 3:**
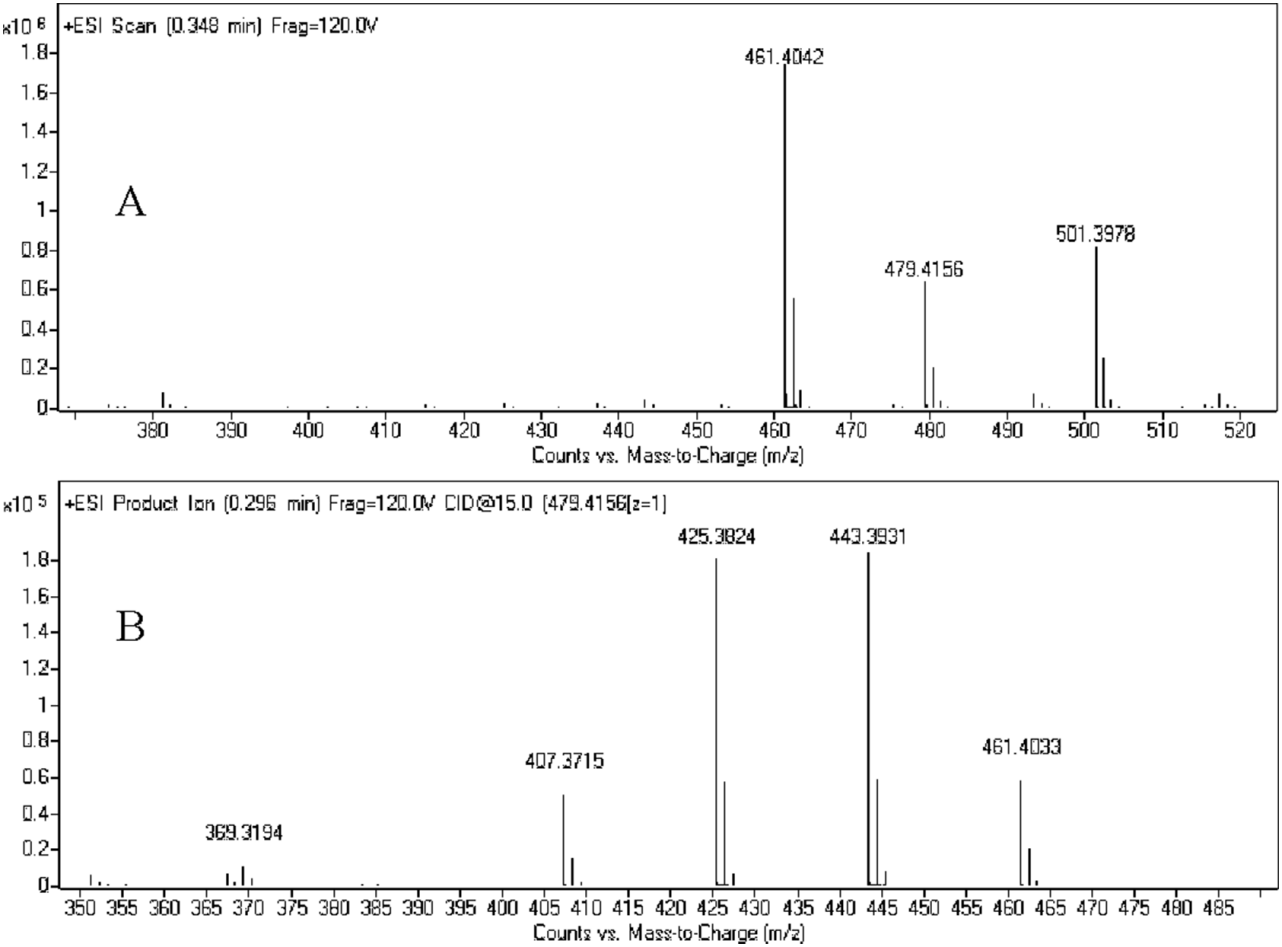
Full-scan mass spectrum (A) and MS^2^ spectrum (B) of 25(R)-OH-PPD.

A comprehensive understanding of the fragmentation behavior of the parent compound to be tested can be very helpful in metabolite identification using LC-Q/TOF. 25(R)-OCH_3_-PPD incubated with hepatic microsomes resulted in seven metabolites was detected from the extracted mass chromatograms, and 25(R)-OH-PPD was identified with the help of standards. Under the experimental conditions, the 25(R)-OCH_3_-PPD molecule (*m*/*z* 493) was detected under positive scan mode (Figure 2A) and *m*/*z* 515 was [M+Na]^+^. In the MS^2^ spectrum (Figure 2B), 25(R)-OCH_3_-PPD displayed diagnostic fragment ions were 475, 457, 443, 425, and 407. Figure 3 showed the spectrum of 25(R)-OH-PPD (*m*/*z* 479) was detected under positive scan mode (Figure 3A) and *m*/*z* 501 was [M+Na]^+^. In the MS^2^ spectrum fragment ions were 461, 443, 425, 407 (Figure 3B).

### LC/MS/MS Analysis by Triple Quadrupole Mass Spectrometry

**Figure 4:**
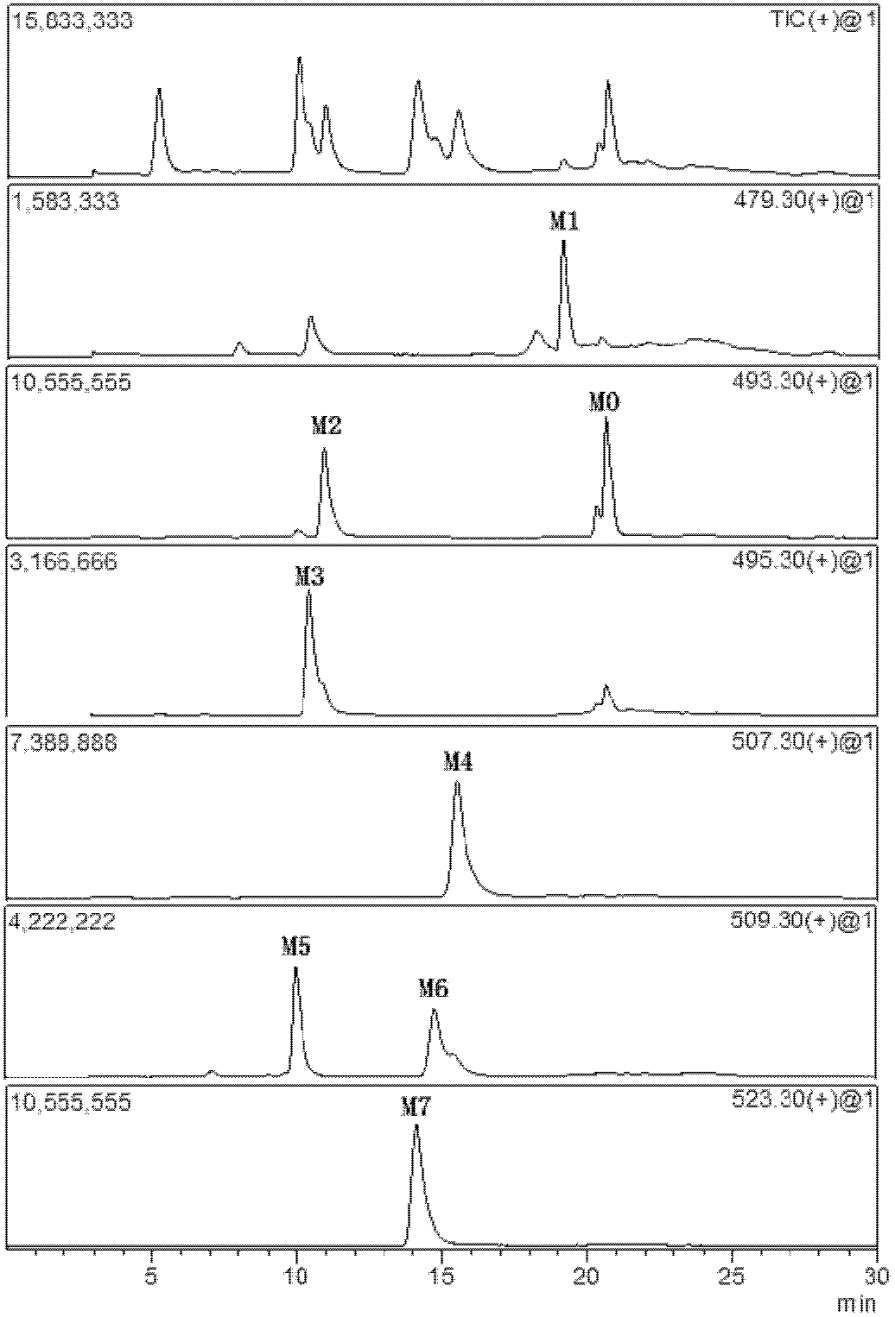
The extracted ion chromatograms of 25(R)-OCH_3_-PPD and its metabolites in HLM with NADPH-regenerating system.

**Figure 5:**
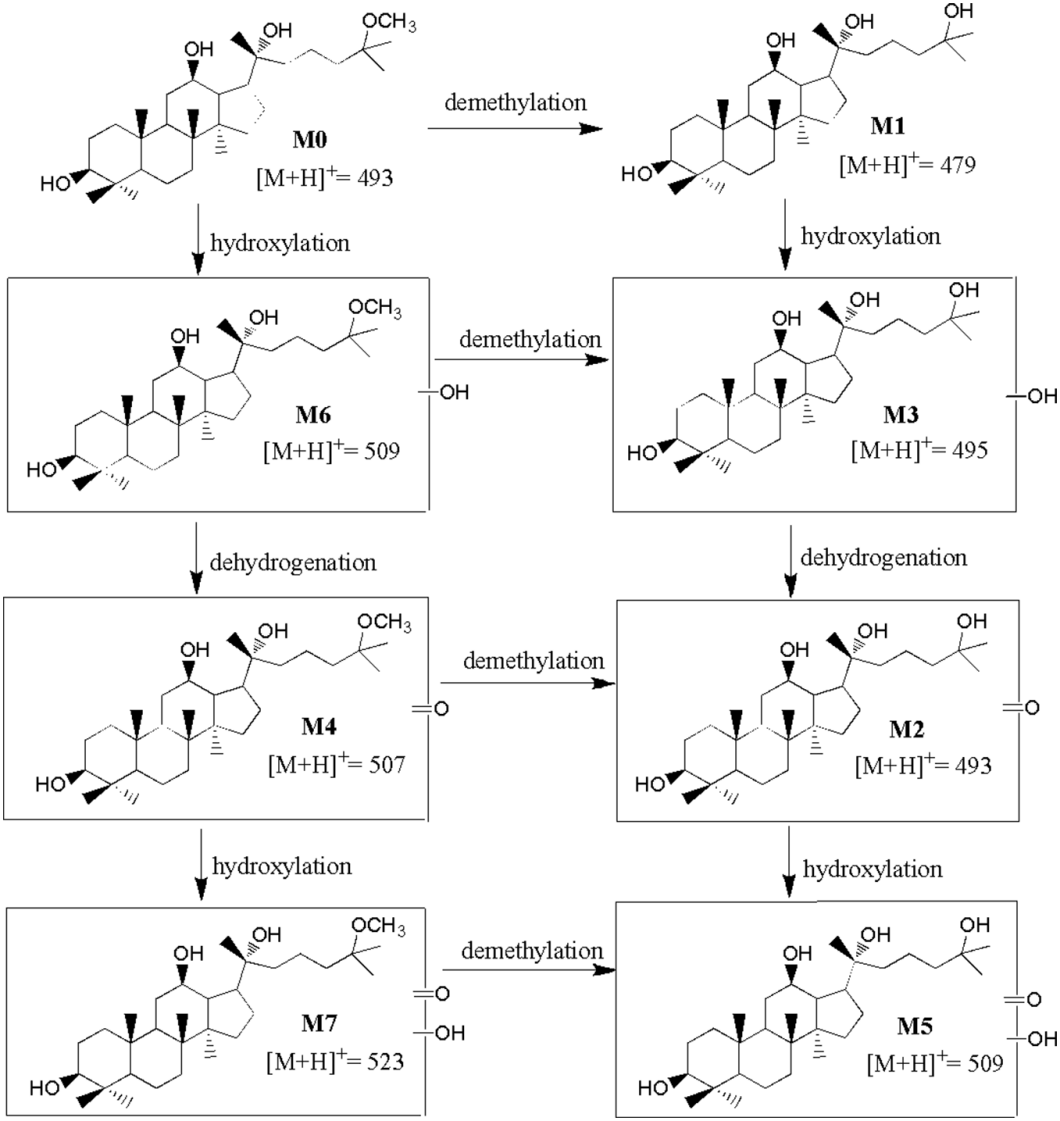
Proposed metabolic pathways of 25 (R)-OCH_3_-PPD in mice, rat, dog, monkey and human microsomes.

When 25(R)-OCH_3_-PPD was incubated without NADPH or microsomes, no metabolites were generated; indicating that there were no chemical reactions under the incubation conditions. Figure 4 showed extracted ion chromatograms of the parent drug and 7 metabolites after incubation in the human liver microsome. Table 1 showed the retention time, MS/MS fragment ions of 25 (R)-OCH_3_-PPD and its metabolites under the present conditions. The phase I metabolite profiles of 25(R)-OCH_3_-PPD were found in all species. Proposed *in vitro* metabolic pathways of 25(R)-OCH_3_-PPD in human and rat microsomes were shown in Figure 5.

**Table 1:** The retention time, MS/MS fragment ions, 25 (R)-OCH_3_-PPD and its metabolites under the present LC-MS/MS conditions.

Metabolite	Description	T_R_ (min)	[M+H]^+^	Formula	Product Ions of [M+H]^+^
M0		20.7	493	C_31_H_56_O_4_	475,457,443,425,407
M1	–CH_2_	19.1	479	C_30_H_54_O_4_	461,443,425,407
M2	–CH_2_+O–2H	11.0	493	C_30_H_52_O_5_	475,457,439,421,403
M3	–CH_2_+O	10.5	495	C_30_H_54_O_5_	477, 459,441,423,405
M4	+O–2H	15.5	507	C_31_H_54_O_5_	489,471,457,439,421,403
M5	–CH_2_+2O–2H	10.1	509	C_30_H_52_O_6_	491,473,455,437
M6	+O	14.8	509	C_31_H_56_O_5_	491,473,459,441,423,405
M7	+2O–2H	14.1	523	C_31_H_54_O_6_	505,487,473,455,437

**Figure 6:**
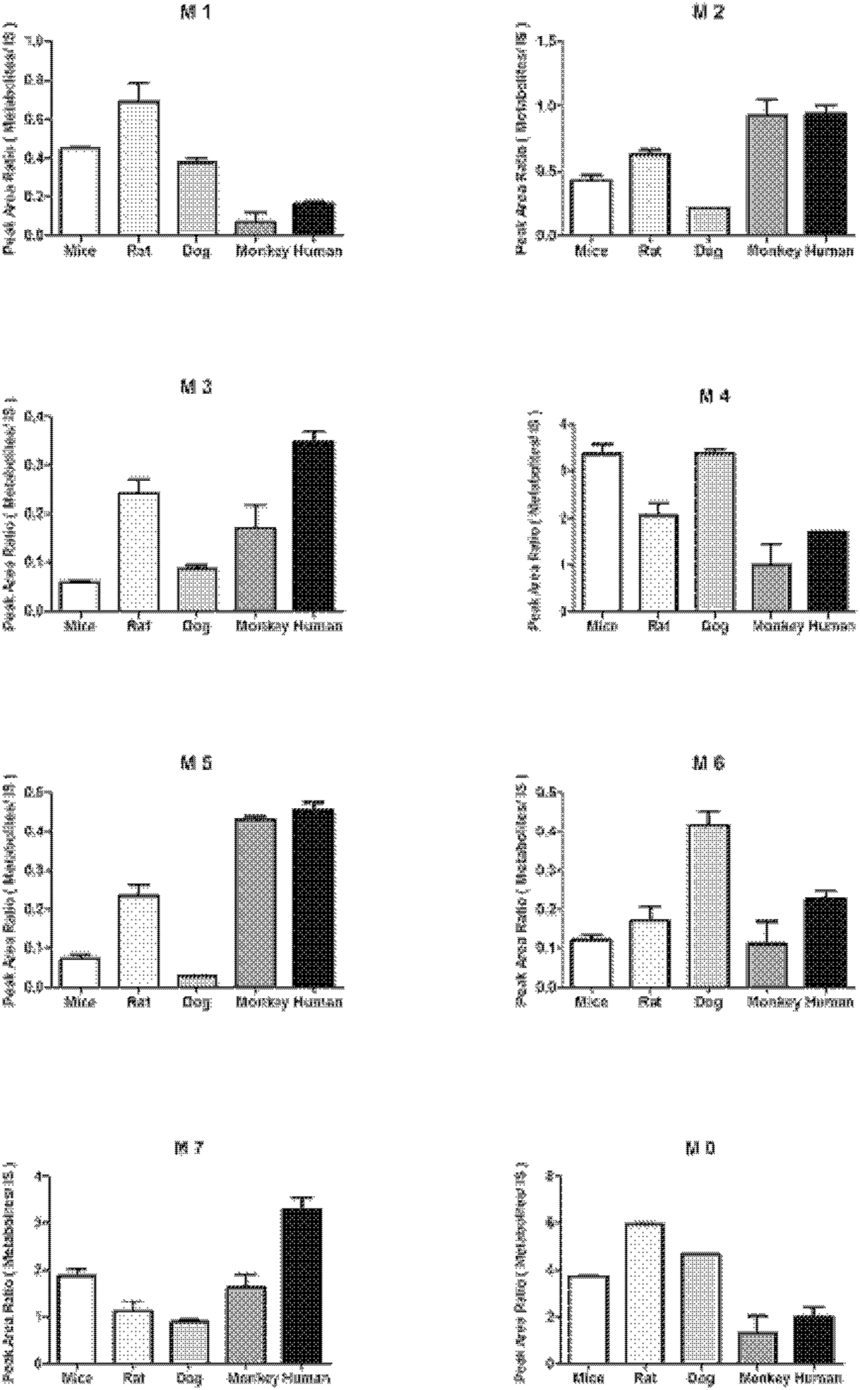
Metabolite formation of 25 (R)-OCH_3_-PPD incubated with NADPH-regenerating system in mice, rat, dog, monkey and human microsomes for 60 min at 37°C. Data are presented as mean ± SD (n = 2).

The quantification for 25(R)-OCH_3_-PPD was performed with the transitions of *m/z* 493.5→425.3 and *m/z* 479.5→425.3 for 20(*R*)-25-OH-PPD. Diazepam with the transition of *m/z* 285.0→154.0 was employed as an internal standard. The assessment of bioanalytical method validation has been described elsewhere [13], [14]. The other metabolites were only semi-quantitated using the ratios of their peak areas relative to that of the internal standard. According to the character of metabolite by MRM in the positive mode was performed using the MS/MS fragment ions, m/z 493.3→439.3 (M2), 495.3→441.3 (M3), 507.3→439.3 (M4), 509.3→455.3 (M5), 509.3→459.3 (M6), 523.3→455.3 (M7).  Mean metabolite amounts found in samples incubated for 60 min are shown in Figure 6. It could be observed that the amount of overall metabolism varied among species.

### Structure Elucidation of 25(R)-OCH_3_-PPD Metabolites in Human, Monkey, Dog, Rat, and Mouse Liver Microsomes

These metabolites were numbered according to the structures were elucidated through their mass spectral fragments and relationship with each other.

25(R)-OCH_3_-PPD (M0) had retention time (T_R_) of 20.7 min and showed protonated molecular ion [M+H]^+^ at *m/z* 493. M0 showed identical fragment ions compared with the parent compound. The chromatography and mass character is the same with 25(R)-OCH_3_-PPD. The MS^2^ spectrum of 25(R)-OCH_3_-PPD showed major product ions at *m/z* 475, 457, 443, 425 and 407. The product ion at *m/z* 443 indicates the loss of methyl group (14 Da).

M1 was observed at 19.1 min with [M+H]^+^ ion at *m/z* 479. The MS^2^ spectrum of M1 showed major fragment *m/z* 461, 443, 425 and 407. The *m/z* value was 14 Da less than that of 25(R)-OCH_3_-PPD, indicating a 25-demethylated metabolite, as was reported in a previous study [14]. Furthermore, comparison with the authentic 25(R)-OH-PPD standard showed identical retention time and MS/MS fragmentation pattern. The lost of 14 Da was used as a key ion for the structural elucidation of 25(R)-OCH_3_-PPD metabolites.

M2 was detected at 11.0 min with [M+H]^+^ ion at *m/z* 493. The product ion scan showed at *m/z* 475, 457, 439, 421 and 403. The 14 Da could not found at MS-MS and indicated lost of methyl group. The *m/z* value of M2 was 2 Da less than that of M3, to deduce the dehydrogenation of M3.

M3 had a retention time of 10.5 min with [M+H]^+^ ion at m/z 495. The MS^2^ spectrum of the M3 [M+H]^+^ ions showed major fragment *m/z* 477, 459, 441, 423 and 405. The product ion *m/z* value was 16 Da higher than that for M1, suggesting hydroxylated product of M1. However, the exact position of M1 hydroxylation could not be determined based on the results of this data. The fragment ion of *m/z* 459 indicated that +16 modification occurred in the dammarane aglycone moiety [16].

M4 was observed at 15.5 min with [M+H]^+^ ions at *m/z* 507. The MS^2^ spectrum of the M4 [M+H]^+^ ions showed major fragment ions *m/z* 489, 471, 457, 439, 421 and 403. The fragment is similar with M6 and the group of methyl was lost. The *m/z* value of M4 was 2 Da less than that of M6, indicating the dehydrogenation of M6. However, the dehydrogenation position cannot be determined by the current data.

M5, 6 were eluted at 10.1 min and 14.8 min with [M+H]^+^ ions at m/z 509. The product ions of M5 at *m/z* 491, 473, 455 and 437 correspond to 16-Da higher forms of the product ions of M2 (475, 457, 439, 421), indicating hydroxylated M2 metabolite. The polarity character of this structure was in accordance with its retention time. The product ions of M6 showed major fragment *m/z* 491, 473, 459, 441 and 423. The *m/z* value was 16 Da higher than that of parent compound, indicating hydroxylated metabollite of 25(R)-OCH_3_-PPD.

M7 was observed at 14.1 min with [M+H]^+^ ion at *m/z* 523. The *m/z* value [M+H]^+^ ions was 16 Da heavier than that for the protonated M4, indicating the introduction of 1 oxygen molecule. The MS^2^ spectra of M7 showed major fragment ions at *m/z* 505, 487, 473, 455 and 437. The product ions correspond to 16-Da higher of the product ions of M4 (*m/z* 489,471,457,439,421), indicating hydroxylation of M4.

### Chemical Inhibition Study in Human Hepatic Microsomes

**Figure 7:**
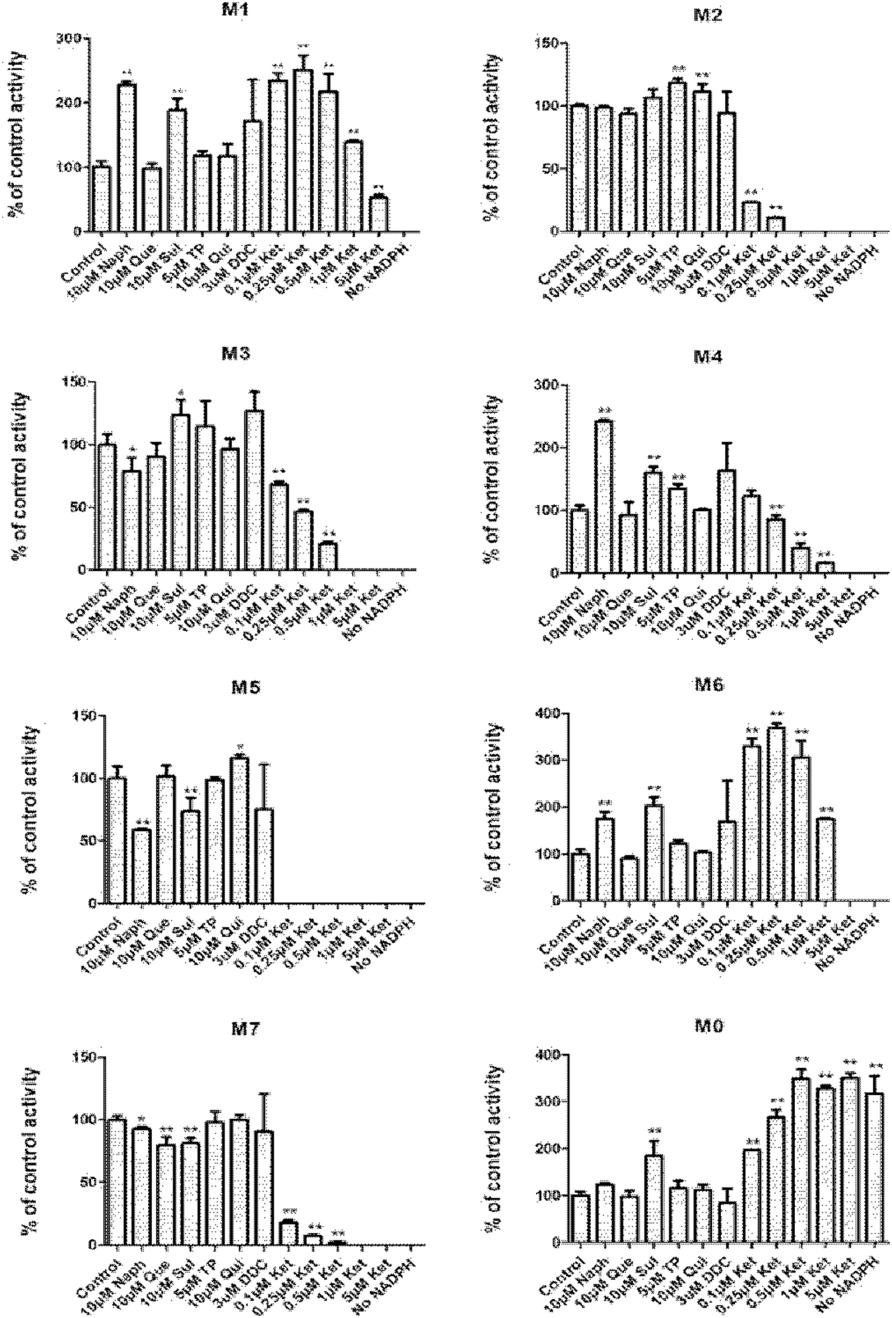
Effects of chemical inhibitors on the formations of metabolites after 25 (R)-OCH_3_-PPD (10 µM) was incubated with HLM (0.25 mg/ml). Data are reported as mean ± SD (n = 3). *P<0.05 and **P<0.01 indicate a significant difference from the control.

**Figure 8:**
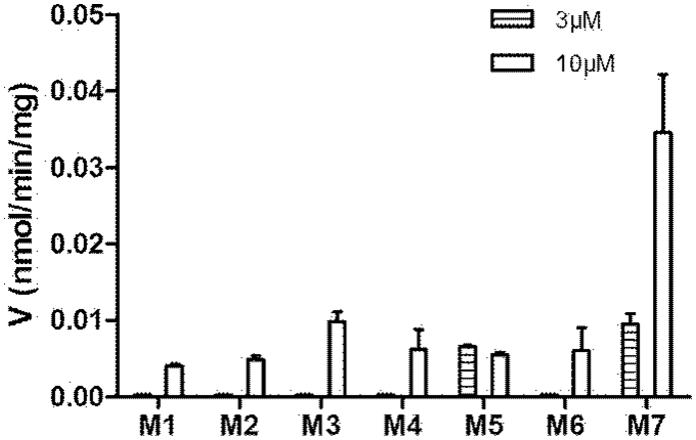
Metabolite M1–M7 formation of 25 (R)-OCH_3_-PPD (3 and 10 µM) incubated with recombinant human CYP3A4 (20 pmol/ml) with NADPH regenerating system for 30 min at 37°C. Each data represents the mean ± SD (n = 3).

Among various specific chemical inhibitors were used, only ketoconazole showed a significant inhibitory effect on the oxidative metabolism of 25(R)-OCH_3_-PPD (Figure 7). All other P450 isoform-specific inhibitors resulted in a negligible effect on the oxygenation metabolism of 25(R)-OCH_3_-PPD. P450 3As were probably the major enzymes responsible for the oxidative metabolism of 25(R)-OCH_3_-PPD. As expected, the oxidative metabolites of 25(R)-OCH_3_-PPD were detected in the incubations only with CYP3A4. The phase I metabolite profile of 25(R)-OCH_3_-PPD in CYP3A4 was shown in Figure 8.

### Enzymatic Kinetics of 25(R)-OCH_3_-PPD Metabolism by HLM

Table 2 showed summary of *K*_m_ and *V*_max_ values for metabolite formation in human liver microsome. Results from the inhibition of an enzymatic process can have serious implications for clinical drug therapy. Preliminary experiments were performed to ensure that the depletion of parent compound was in the linear range of both reaction time and protein concentration of microsomes. The apparent *K*_m_ for M2, M5, M7 was<5 µM under our conditions; but the value *K*_m_ for M1, M3, M4, M6 was 19–30 µM, and the *V*_max_ value ranged from 11.3 (M2) to 165 (M4) pmol/min/mg protein.

**Table 2:** Kinetics parameters of formation of M1–M7 from 25 (R)-OCH_3_-PPD in human liver microsome.

Parameters	M1	M2	M3	M4	M5	M6	M7
**K_m_ (µM)**	26.4	1.23	19.8	19.0	1.58	24.5	4.92
**V_max_**(**pM/min/mg**)	42.1	11.5	23.2	165	11.4	113	73.2
**V_max_/K_m_** **µl/**(**min/mg**)	1.59	9.35	1.17	8.68	7.21	4.61	14.9

## Discussion

The possible cancer preventive activity of *Panax notoginseng* is receiving more and more attention. Information on the metabolism of *Panax notoginseng* components is important for understanding the biological effects of *Panax notoginseng*. Some reports concerned the structure-activity relationship and substrate-dependent phenomena in effects of Ginsenosides on P450 Enzymes [15], [16]. Previously, we reported the pharmacokinetics of 25(R)-OCH_3_-PPD in rat. Thus far, the differences in the metabolic pathways and behaviors of 25(R)-OCH_3_-PPD between human and common experimental animals have not been revealed. Hence, in this study a comparison of metabolic profiles, enzymes involved, and catalytic efficiency for 25(R)-OCH_3_-PPD metabolism in liver microsomes from different species was performed.

The products of 25(R)-OCH_3_-PPD biotransformation with liver microsomes *in vitro* were first analyzed using LC–TOF–MS. We speculated structures of the metabolites in positive-ion mode on the fragment rules. Then, the parent compound and M1–M7 were quantified by LC/MS/MS performed on Shimadzu LC-MS/MS-8030 following positive mode electrospray ionization.

A total of 7 metabolites were identified based on the retention times, molecular weights, parent ions and fragment ions which are presented in Table 1. The proposed metabolic pathways of 25(R)-OCH_3_-PPD in liver microsome were shown in Figure 5. These metabolites may be classified into three types: demethylation (M1, M2, M3 and M5), dehydrogenation (M2 on base of M3), (M4 on base of M6) and hydroxylation (M6 on base of M0, M3 on base of M1, M5 on base of M2, M7 on base of M4). Future works need to confirm whether demethylation was formed from M6 to M3, M4→M2 and M7→M5. The methyl group was easily removed by phase I enzymes and hydroxylation derivatives were the main metabolites.

We were only able to investigate the metabolites M2–M7 by semi-quantitated method due to the lack of authentic standards. The intrinsic clearance (*V*_max_/*K*_m_) ranked as follows: M3< M1< M6< M5< M4< M2< M7 (Table 2). It was necessary to address the observed discrepancies of catalytic efficiencies among the animal liver microsomes. The study revealed a comparative metabolism of 25(R)-OCH_3_-PPD in liver microsomes from mouse, rat, dog, monkey, and human. The metabolites were similar across all animal species, with a few distinct differences in amount (Figure 6).

Metabolite M1 was the dominant metabolite from the rodent species after incubation of 25(R)-OCH_3_-PPD with microsomes, was found in an intermediate relative amount in dog, and was a minor metabolite in monkey and human microsomes. In addition M3 and M5 were the major product of 25(R)-OCH_3_-PPD metabolism in HLM, but a very minor product of incubation from microsomes of MLM and in dog. However, the more metabolites M4 and M7 were found in all species. In particular, M5 was the major metabolite in CyLM and HLM under our experimental parameters, while this compound was minor in all other species, with the smallest amount produced from DLM incubation.

For further exploration of the similarity of the enzymes responsible for the metabolism of 25(R)-OCH_3_-PPD from various species, chemical inhibition studies with selective general inhibitors were administrated. Ketoconazole inhibited the formation of M2, M3, M4, M5, M7 and showed concentration dependent (Figure 7). Ketoconazole inhibited the formation of M5 completely at concentration of 0.1 µM. Ketoconazole (0.1–1 µM) activated formation of M1 and M6, but at 5 µM inhibited the formation of M1 and M6. The reason is that M1 and M6 were not sensitive to ketoconazole.

Both chemical inhibition studies and recombinant enzyme screen results showed that CYP3A4 was the major P450 isoform in formation of all oxidative metabolites of 25(R)-OCH_3_-PPD and M7 was with the largest amount among the metabolites. Previous work by Li et al [16] demonstrated that CYP3A4 contributed to the formation of all oxidative metabolites of Rh2. Rh2 is also a dammarane-type triterpenoid saponin formed by a glucose sugar moiety glycosidically bonded to the hydroxy group at the C-3 position of 20(S)-protopanaxadiol (PPD). The inhibition of the CYP3A4 catalytic activity by 25(R)-OCH_3_-PPD was NADPH and concentration-dependent, which supplies a possible explanation for the low absorption of 25(R)-OCH_3_-PPD in rats in our previous research.

## Conclusions

This report gave an in-depth exploration of 20(R)-25-OCH_3_-PPD metabolisms in liver microsomes from mouse, rat, dog, monkey, and human. A qualitative profiling by LC/TOF-MS led to the qualitative detection of seven metabolites, including hydroxylation, dehydrogenation and O-demethylation. LC/MS/MS analysis was used to quantify parent and metabolites in different microsomes with NADPH-dependent. CYP3A4 was the predominant enzyme involved in the oxidative metabolism. These data are fundamental toward future work in 20(R)-25-OCH_3_-PPD chemoprevention.
